# A Study of Normal Renal Dimensions at Ultrasonography and Their Influencing Factors in an Indian Population

**DOI:** 10.7759/cureus.40748

**Published:** 2023-06-21

**Authors:** Rana P Singh, Arshad Jamal

**Affiliations:** 1 Department of Urology, Rajendra Institute of Medical Sciences, Ranchi, IND

**Keywords:** indian population, parenchymatous thickness, renal width, renal length, chronic renal failure, renal dimension

## Abstract

*Background: *Kidney dimensions play one of the most vital roles in the diagnosis and identification of any renal disease. Renal dimensions are generally used in clinical practices to determine the size of the kidney as well as correlate with renal function to have a better understanding of acute and chronic renal diseases. This study aimed to find out the normal renal dimensions with the help of ultrasonography and their impact on the Indian population.

*Methods:* Renal dimensions, which include parenchymatous thickness and length as well as the width of about 60 healthy adult Indian populations, were estimated with the help of sonography, and a detailed study has been performed on the difference observed based on age, sex, height, weight, body mass index, and body surface area.

*Results: *There was no particular difference found on the basis of width and length between the left and right kidneys; however, the parenchymal thickness between the left and right has been shown to have a significant difference. The mean width, length, and parenchymal thickness were 4.6 ± 0.43, 9.64 ± 0.62, and 2.03 ± 0.1 cm, respectively. While doing estimation based on gender, it has been observed that there is a noticeable difference in width but no difference in height or parenchymal thickness. A significant diversity has been observed in patients in age groups above 49 compared to other age groups. A positive correlation with body weight, body height, and body mass index has also been observed in some cases.

*Conclusion:* The given study has attempted to define the standard reference for renal dimension in the Indian census. The observations made in the given study demonstrated the possibility of renal dimensions being smaller in the Indian population in contrast to those of the Western population, which are much larger.

## Introduction

The kidney is one of the most vital organs in our body, and unilateral and bilateral changes seen in the kidney are vital parameters in the clinical management and evaluation of patients who have renal diseases [1]. Rising incidences of kidney disease in India can be responsible for impending threats to healthcare and the economy in future years [2]. Renal ultrasonography is proven to be a secure, affordable, reliable, and easily attainable way of kidney imaging, which is presently used for assessment and follow-ups of patients with renal cystic diseases and arterial stenosis, chronic kidney disease, kidney stones, vesicoureteral reflux, kidney tumors, congenital anomalies, and transplants, and recurrent urinary tract infections, both in infants as well as adults [3,4]. A review of the literature reveals differences in renal volume and size with respect to gender, age, BMI, and various disorders. The size of the impaired kidney will rise if any mass develops within it. The kidney can enlarge to the size of a football once a cyst begins to form. Significant co-morbid conditions impacting renal size include interstitial nephritis, diabetes, lupus nephritis, hypertension, and glomerulonephritis. A reduction in blood flow could be one of the possible reasons for the shrinkage in kidney size [5,6].

Ultrasound (USG) is among the most commonly used imaging techniques and helps in the estimation of kidney length. In addition, the imaging technique, multi-detector computerized tomography (MDCT), is employed to evaluate the sizes of both kidneys [7]. Sonography can be performed to obtain an estimation of renal size by measuring renal length (RL) and kidney volume (KV), cortical thickness, or volume. It has essentially supplanted the intravenous pyelogram as the first modality for the examination of the kidneys since it provides great anatomical details, does not expose patients to radiation, does not use contrast agents, and does not demand any special patient preparation procedure [8]. When deciding whether to opt for a renal biopsy, a kidney transplant, or avoid immunosuppressive medication, renal size is crucial. Magnetic resonance imaging (MRI) and computed tomography (CT) are other imaging modalities for measuring kidney diameters [9-11].

Animal studies on renal length and cortical thickness have yielded much information on these variables [12,13]. Only a few studies in humans have attempted to assess these characteristics in individuals without renal illness [14,15]. Unfortunately, several of the adults involved in earlier trials had conditions, including diabetes and hypertension, that could have a negative impact on the kidneys [16]. The majority also considered blood creatinine as the sole indicator of normal renal function, omitting the evaluation of daily urine protein excretion and urinary sediments to eliminate glomerular structural problems [12]. Even if serum creatinine is within the normal range, it is not a reliable indicator of renal function and does not eliminate the occurrence of renal disease. This study aims to estimate the average renal dimension in the human population and their relative relations with weight, height, age, body mass index (BMI), and body surface area (BSA).

## Materials and methods

A cross-sectional study was conducted at the Department of Urology, Rajendra Institute of Medical Sciences (RIMS), Ranchi, India, with 60 patients over the age of 21. Informed consent was obtained in writing, according to the ethical protocol, from all participants referred for an ultrasound for non-renal indications. Among the total sample population, 19 (32%) were referred for biliary or liver indications, 11 (19%) for musculoskeletal ultrasounds, 13 (23%) for neck ultrasounds, and 17 (26%) for miscellaneous indications.

Inclusion criteria

Patients with eGFR calculated using a modification of diet in renal disease (MDRD) formula > 30 ml/min/m2, serum creatinine within the last six months ≤ 1.5mg/dl, Normotensive during ultrasound examination (diastolic BP less than 90 mmHg and systolic BP less than 140 mmHg), and no other diseases causing renal damage were included in this study. A total of 60 patients were included in the study after fulfilling the inclusion criteria.

Exclusion criteria

Patients who already had symptoms suspecting renal pathology or other medical histories of renal canaliculi or kidney stone disease, patients who have undergone abdominal surgery for renal infections, patients with a history of hypertension, diabetes, or both, and pregnant women were excluded.

During the conduction of the sonograms, one patient with asymptomatic hydronephrosis, one with a horseshoe kidney, two with unilateral kidneys, one with undetected asymptomatic renal calculi, one patient with undetected pregnancy, one with renal malposition, three with renal pole cysts, and two with polycystic kidney disease were excluded from the study.

Statistical analysis

The spread of the data and its tendency has been calculated using descriptive statistics. With the help of the unpaired t-test, sex-related differences in the mean of varied renal parameters were studied. However, the differences between right and left renal parameters were studied with the help of the paired Student t-test. An association test was performed to determine the Pearson correlation coefficient between the renal lengths and anthropometric variables. Dissimilarities in right and left renal dimensions among varied age brackets were contrasted with a one-way variance analysis. Following this, a post hoc Tukey test was performed. With the help of anthropometric values, renal indices were calculated for normalizing renal lengths (L/BSA and L/BMI).

All patients underwent an ultrasound of both kidneys using the Toshiba Power Vision 6000, Tokyo, Japan, an ultrasound device with a 3.5 MHz convex transducer. The techniques and parameters that were set to get the estimated dimensions were as follows: Renal width (RW), Renal length (RL), and Parenchymal thickness (PT). Patients were asked to void their bladders before the examination because it can lead to a hydration-associated increase in renal length. The height and weight of the patients were calculated immediately after the ultrasound examination. A stadiometer has been used to measure the height of the patients without using footwear. BSA was computed using the formula of BSA = Wt x Ht 36. BMI was computed using the formula BMI = Wt/Ht2. Here, Ht is the height measured in ‘m’, and Wt is the weight measured in ‘kgs.’

The study was approved by the institutional ethics review board (Institutional Ethics Committee RIMS, Ranchi; Memo Number: 78, IAEC/IEC RIMS, Ranchi).

## Results

Sixty patients were analyzed among 123 renal units. Of the patients, 39 and 21 were males and females, respectively, between the ages of 21 and 72 years. Age distribution was carried out as follows: 12 (21-29 years), 28 (30-39 years), 35 (40-49 years), and 25 (> 49 years). The range of eGFR values was 44 to > 90 ml/min/m2, and the range of creatinine values was 0.4 to 1.5 mg/dl. The mean length was 9.63 ± 0.64 cm, with a 7.6-12.5 cm range. The mean parenchymal thickness was 2.05 ± 0.1 cm, and the mean renal width was 4.6 ± 0.41 cm.

Correlations between Left Renal Length (LL) and Right Renal Length (RL) with body indices, viz., height, weight, BMI, and BSA, were estimated with the help of Pearson’s correlation coefficient. This exhibited a correlation of BSA (r = 0.33 and r = 0.32, respectively, for the right and left kidney) and renal length with body weight (r= 0.31 and r = 0.32, respectively, for the right and left kidney). However, a weak positive correlation was seen with BMI (r = 0.23 and r = 0.20, respectively, for the right and left kidneys) and body height (r = 0.19 for both kidneys). Multiple regressions performed gave the following equations for renal length determination from anthropometric parameters:

Left renal length (LL) (cm) = 0.03 Weight + 6.95 + 1.02 Height, Right renal length (RL) (cm) = 0.04 Weight + 6.45 + 1.12 Height, Where height is in ‘m’ and weight is in ‘kg’.

## Discussion

It has been observed from most of the studies about the renal dimension that the result in the Caucasian population is quite in contrast to that of the Indian population [3]. The renal dimension in the Indian population is smaller as compared to that of Caucasia, Korea, Malaysia, Brazil, Nigeria, and Jamaica, but its relationship with differences in weight, height, BMI, BSA, and other anthropometric factors between races is questionable [1,4,5,11]. The possible reasons for such differences in size could be attributable to the fetal environment, nucleotide polymorphisms that influence nephron endowment, and varying lifestyle factors such as obesity and a high-salt diet, among others. Furthermore, it was observed that sex does not have any deciding factor in the relative renal length; thus, the height difference is a more reliable parameter when compared with the absolute renal length [1]. 

The dimension of the kidney is one of the most important factors in the determination of renal diseases [17]. The size of an average right kidney is larger than that of a left kidney, with an average of 10.0-12.4, which varies among the different populations with a cut-off renal length of 9 cm to highlight irreversible renal diseases. The presence of a larger right kidney could act as a clinical clue to diabetes mellitus, acute nephritis, acute renal failure, pregnancy, or even compensatory hypertrophy of the kidney. Therefore, differences in right and left renal lengths can serve as a basis for making an informed clinical decision. In this study, the mean length of the left kidney was found to be 98.90 ± 9.8 mm. The renal lengths were found to be highest among males (103 ± 9.37) and females (100.0 ± 6.43) of the age group 21-29 (Table 2). Similar findings were reported in certain other Indian studies [14-16].

A decline in parenchymal thickness and renal length was observed in people over 70 years of age only. Gender differences were also observed between renal lengths, where high values were seen for both the left (103.6 ± 7.08) and the right kidneys (98.7 ± 7.08) among males (Table 1). The Pearson Correlation coefficient of RL was 0.31, while LL was 0.68, right renal breadth (RW) was 0.038, left renal breadth (LW) was 0.037, right renal parenchymal thickness (RCT) was 0.098 and left renal parenchymal thickness (LCT) was 0.088. A consistent observation throughout many studies was that of larger kidney sizes among males [18,19]. In this study, the right kidney length (28 counts) was found to be larger and smaller than 90 mm when compared with the left kidney (18 counts). However, the LL (13 counts) was found to be larger (> 111 mm) than the right kidney (2 counts) (Table 3). But apparently, there were no sex-related differences in renal length. Weight and height were independent indicators of renal size (Tables 4, 5). This is evident from Table 4, where the renal length values in males were seen increasing according to height. But there were a few fluctuations in numbers evident in the case of females. In Table 5, the renal length, according to weight, fluctuated for both males and females. BSA is the most sensitive indicator in this study, a finding that can logically be proven to relate directly to body size.

**Table 1:** Renal length by gender

Renal dimension	Female	Male
Left kidney	98.5 ± 9.95	103.6 ± 7.08
Right kidney	94.5 ± 8.57	98.7 ± 7.08

**Table 2:** Renal length by age

Age group in years	Male	Female
21-29 (n=12)	103 ± 9.37	100.0 ± 6.43
30-39 (n=28)	98.6 ± 9.28	94.6 ± 8.42
40-49 (n=35)	99.2 ± 8.81	94.7 ± 9.18
>49 (n=25)	98.1 ± 11.49	94.4 ± 8.05

**Table 3:** Renal length distribution by size

Kidney length in mm	Right kidney	Left kidney
<91.0	28	18
91.0 - 100.9	47	35
101 - 110.9	21	32
>111	2	13

**Table 4:** Renal length by height

Height in cms	Renal length in male	Renal length in female
< 150 (n = 6)	94.5 ± 10.53	90.0 ± 6.78
150.1 - 160 (n = 57)	97.2 ± 9.75	94.2 ± 8.71
160.1 - 170 (n = 34)	102.6 ± 8.47	97.2 ± 7.72
>170 (n = 3)	106.7 ± 7.12	103.2 ± 1.71

**Table 5:** Renal length by body weight

Body weight (kg)	Renal length in male	Renal length in female
<50 (n = 5)	94.7 ± 6.93	95.2 ± 3.43
50 - 60 (n = 51)	98.2 ± 10.18	94.5 ± 9.06
60.1 - 70 (n = 39)	100.8 ± 8.93	96.2 ± 8.32
>70 (n = 5)	99.2 ± 12.83	97.6 ± 0.37

**Table 6:** Renal length by BMI

BMI (kg/m2)	Renal length in male	Renal length in female
<20 (n = 4)	110.8 ± 5.65	102.4 ± 7.99
20.1 - 24 (n = 45)	99.0 ± 9.00	96.5 ± 6.93
24 - 28 (n = 48)	98.1 ± 9.99	93.5 ± 9.54
>28 (n = 3)	94.3 ± 13.17	95.6 ± 8.57

Renal length is considered a crucial renal parameter that significantly correlates with body height and weight, among which weight indicates a stronger relationship [20,21]. These variables were recognized as independent indicators of renal length in our multivariate model. The findings infer that physique and body build are major factors in predicting renal sizes among healthy people, even though some parameters might have more impact than others. It is the amalgam of these standard measurements that helps determine kidney size in healthy humans [21]. Although a significant relation depicts that the length of the kidney increases with an increase in the height of the left kidney only, it was also observed that renal length heightened with age up to 50 years in both males and females. This then gradually decreased from the sixth decade onward. In the case of BMI, it was observed that the renal length in males (110.8 ± 5.65) was the highest in males with BMI less than <20.0 and in females (102.4 ± 7.99) of the same group (Table 6). As a result, this establishes the existence of a negative correlation between kidney length and age and a positive correlation between BMI, kidney length, and height [22].

Due to the difficulty in detecting the maximum bipolar length plane and its dependency on the operator, ultrasound can cause a glitch in measuring the actual size of the kidney. Thus, it is possible that certain measurements cannot be constructed parallel to the kidney axis, in contrast with different radiological techniques for kidney length measurement. Radiological methods are essential to predicting errors, with CT being the least witnessed [23-27]. The study is expected to provide near-actual measurements of kidney dimensions from the Indian census.

The study’s limitations include the fact that it only dealt with non-renal pathologies, i.e., a healthy population. Furthermore, the low sample size questions the generalizability of the results. Another drawback of our study is that only linear measurements of the kidney have been made, and we did not have any non-volumetric comparisons. The findings of our study indicate that there is a need for future studies with a larger sample size and incorporating individuals from various Indian ethnicities.

## Conclusions

The main goal of this research was to prospectively produce normal data for renal dimensions in the general population using sonography. The result of this study provides valuable data for establishing reference values for kidney size in the Indian population using ultrasonography. The data presented in this work showed that height difference is a more reliable parameter when compared with the absolute renal length values presented in previous literature. Our study showed that renal breadth tends to be affected by gender as opposed to renal length. No known subjects had any conditions like diabetes or hypertension, as these conditions and their treatments can affect kidney sizes. In conclusion, this study provided data for normal sonographic renal dimensions in the Indian population and also derived equations to predict the expected normal renal lengths from anthropometric values.
